# Stakeholder perspectives and requirements to guide the development of digital technology for palliative cancer services: a multi-country, cross-sectional, qualitative study in Nigeria, Uganda and Zimbabwe

**DOI:** 10.1186/s12904-020-00694-y

**Published:** 2021-01-04

**Authors:** Kennedy Bashan Nkhoma, Bassey Ebenso, David Akeju, Samuel Adejoh, Michael Bennett, Mike Chirenje, Adlight Dandadzi, Elizabeth Nabirye, Elizabeth Namukwaya, Eve Namisango, Kehinde Okunade, Omolola Salako, Richard Harding, Matthew J. Allsop

**Affiliations:** 1 Florence Nightingale Faculty of Nursing Midwifery and Palliative Care, Cicely Saunders Institute, King’s College London, London, UK; 2 Nuffield Centre for International Health and Development, Leeds Institute of Health Sciences, University of Leeds, Leeds, UK; 3 Department of Sociology, University of Lagos, Lagos, Nigeria; 4 Academic Unit of Palliative Care, Leeds Institute of Health Sciences, University of Leeds, Leeds, UK; 5 Clinical Trials Research Centre, College of Health Sciences, University of Zimbabwe, Harare, Zimbabwe; 6 Department of Internal Medicine, Makerere University, Kampala, Uganda; 7 African Palliative Care Association, Kampala, Uganda; 8 College of Medicine, University of Lagos, Lagos, Nigeria; 9 Department of Radiation Oncology, Lagos University Teaching Hospital, Lagos, Nigeria

## Abstract

**Introduction:**

Coverage of palliative care in low and middle-income countries is very limited, and global projections suggest large increases in need. Novel approaches are needed to achieve the palliative care goals of Universal Health Coverage. This study aimed to identify stakeholders’ data and information needs and the role of digital technologies to improve access to and delivery of palliative care for people with advanced cancer in Nigeria, Uganda and Zimbabwe.

**Methods:**

We conducted a multi-country cross-sectional qualitative study in sub-Saharan Africa. In-depth qualitative stakeholder interviews were conducted with *N* = 195 participants across Nigeria, Uganda and Zimbabwe (advanced cancer patients *n* = 62, informal caregivers *n* = 48, health care professionals *n* = 59, policymakers *n* = 26). Verbatim transcripts were subjected to deductive and inductive framework analysis to identify stakeholders needs and their preferences for digital technology in supporting the capture, transfer and use of patient-level data to improve delivery of palliative care.

**Results:**

Our coding framework identified *four* main themes: i) acceptability of digital technology; ii) current context of technology use; iii) current vision for digital technology to support health and palliative care, and; iv) digital technologies for the generation, reporting and receipt of data. Digital heath is an acceptable approach, stakeholders support the use of secure data systems, and patients welcome improved communication with providers. There are varying preferences for how and when digital technologies should be utilised as part of palliative cancer care provision, including for increasing timely patient access to trained palliative care providers and the triaging of contact from patients.

**Conclusion:**

We identified design and practical challenges to optimise potential for success in developing digital health approaches to improve access to and enhance the delivery of palliative cancer care in Nigeria, Uganda and Zimbabwe. Synthesis of findings identified 15 requirements to guide the development of digital health approaches that can support the attainment of global health palliative care policy goals.

**Supplementary Information:**

The online version contains supplementary material available at 10.1186/s12904-020-00694-y.

## What is already known?

- Palliative care is a critical and essential component of care which requires further development to support increasing numbers of people with cancer in sub-Saharan Africa
- Digital technology is used widely across palliative care services in sub-Saharan Africa, but it is not clear how it can be leveraged to support service delivery and the development of an underpinning evidence base

## What are the new findings?

- Unmet data and information needs were identified across patients, caregivers, health professionals and policymakers in Nigeria, Uganda and Zimbabwe, with digital technology viewed as an acceptable approach to enhancing existing provision of palliative care
- 15 key requirements of digital technologies were synthesised from across stakeholder groups that can be used to guide the future development and evaluation of digital technology approaches in palliative cancer care

## What do the new findings imply?

- User engagement across diverse stakeholder groups is feasible and provides novel insights to inform technology design for palliative cancer care in sub-Saharan Africa which should be continued throughout subsequent development and implementation of digital technology approaches
- Working with donors and private industry, governments and policymakers in sub-Saharan Africa are best-placed to ensure resultant digital technologies for palliative cancer care are interoperable, maintain privacy and confidentiality of data and adhere to emerging governance frameworks.

## Introduction

Global cancer prevalence is increasing disproportionately in low and middle-income countries (LMICs). By 2060 an estimated 16 million people with cancer will die annually with serious health-related suffering, a 109% increase on current figures, with the fastest rise occurring in low-income countries (i.e. a 400% increase) [1]. Increased ageing, high residual burden of infectious agents (such as HIV/AIDS, human papillomavirus, and hepatitis B virus) and adoption of lifestyle factors (e.g. smoking tobacco and excessive alcohol intake) are the main drivers of this increase [2, 3]. Despite the rising number of cases and unmet need, cancer and its treatment has long been neglected in global health [4].

It is estimated that 80% of cancers in the SSA are incurable and advanced at the time of detection and diagnosis due to late clinical presentation and poor access to prevention and treatment facilities [5]. For those with cancer, palliative care aims to prevent and relieve physical, emotional, social, and spiritual suffering potentially at any stage of the disease [6]. This care is crucial to support patients who are often experiencing high levels of pain and symptom burden alongside reductions in social functioning [7], presenting significant physical and emotional challenges for their caregivers [8]. Palliative care provision using a person-centred approach is a vital and fundamental component of the basic and essential services within universal health coverage (UHC) [9]. UHC requires that all individuals and communities receive the health services they need without suffering financial hardship (affordable services). This includes the full spectrum of essential, quality health services, from health promotion, prevention, treatment, rehabilitation, and palliative care. Palliative care is a human right [10], enabling patients and families to live well with progressive illness, improving their outcomes, and saving costs by reducing unplanned admissions and futile treatments [11–15]. Palliative care remains a critical and essential component of care, and strong body of evidence demonstrates its effectiveness and cost-effectiveness [12, 13, 16].

Through research, consultation with advocacy organisations and policy involvement our team have been developing approaches to gathering data that can help shape provision of palliative care for cancer patients and their caregivers [17]. We identified widespread use of digital technologies by patients and palliative care health professionals in the region. Digital technology, or digital health in its broadest sense, is defined as “the use of information and communications technology in support of health and health-related fields” [18]. Development of approaches that capitalise on digital technology is a high priority for service providers [5, 19] and the WHO has recommended their use as an approach that can support health systems strengthening in LMICs [18]. Primary research is essential to understand the best ways to collect data via digital technologies (such as mobile phones), to ensure alignment with needs of patients, caregivers, health professionals and policymakers and to incorporate technology into routine practice.

Researchers and clinicians are finding new ways to harness digital technologies to ensure good linkage with patients and caregivers [20]. Through use of digital technologies, patients and caregivers can communicate in real time with palliative care services (e.g. symptom reporting and self-management support and advice, or videoconferencing for consultations and discussions). It can also be used to enhance communication between health professionals, such as conducting multidisciplinary meetings, discussion of complex cases or delivering mentorship via teleconference [20]. The application of digital technologies may improve access to care (irrespective of location), especially when face-to-face contact is not feasible or is costly [21]. The use of digital technologies to implement clinical consultations such as videoconferencing has been found to be an appropriate and suitable model of care in particular if patients and caregivers are actively involved in decision making [22]. In recent years, scale-up and implementation of digital interventions have been developed in, for example, child and maternal health, and provided evidence of being able to support the operationalisation and achievement of universal health coverage (UHC) [23]. In the context of SSA, increasing growth of mobile phone ownership and supporting infrastructure provide opportunities for digital health. In SSA, around half of all people own a mobile phone, one third of which are smartphones [24]. Furthermore, there are ongoing efforts to develop the supporting infrastructure (with 70% coverage of 3G networks and 34% with 4G across the SSA region), alongside targeting the affordability, content and services, and consumer readiness for digital technologies [25]. Research in the development of digital technologies for palliative care in SSA is also emerging, with existing literature reviews, surveys of digital technology use and pilot studies [17, 26, 27]. However, there is a lack of an underpinning evidence base to inform the design and development of interventions using digital health components in this context. This study was conducted in Nigeria, Uganda and Zimbabwe, where previous high levels of mobile phone utilisation have been identified across palliative care providers [17]. However, there is varying supporting policy for cancer and digital health in these three countries, with a digital health policy present only in Uganda [28]. This study aimed to identity and engage key stakeholders (patient, caregivers, health professional and policymakers) across the health systems of Nigeria, Uganda and Zimbabwe to identify and define optimal mechanisms through which digital technologies can be used to improve access to and delivery of palliative cancer care in SSA.

## Methods

The study protocol has been published previously [28] which includes an overview of the context to palliative care access and its level of development in Nigeria, Uganda and Zimbabwe, alongside the policy context in each country. This cross-sectional study used qualitative in-depth face-to-face interviews, and has been reported in line with the COnsolidated criteria for REporting Qualitative research (COREQ) checklist [29].

### Setting

Participating sites were public facilities and non-profit, non-governmental organisations. In Uganda, we collected data at four sites: Uganda Cancer Institute, Makerere Palliative Care Unit at Malago Hospital, Kawempe Home Care and Hospice Africa Uganda. In Zimbabwe, we recruited at Chitungwiza Hospital, Parirenyatwa Group of Hospitals, and Island Hospice and Health Care. In Nigeria, we recruited at Lagos University Teaching Hospital and Sebeccly Cancer Care and Support Center in the Lagos metropolis.

### Study participants

We sampled four stakeholder populations:

#### Patients with advanced cancer

Inclusion criteria were patients (*n* = 20 in each country) aged at least 18 years, aware of their diagnosis of advanced cancer and receiving palliative care. A purposive sampling frame identified potential participants with variation according to sex, income, family size and support, employment, cancer type, and duration of time since referral to palliative care.

#### Family/primary caregivers

Caregivers (*n* = 15 in each country) of patients who met the patient criteria above. Inclusion criteria was at least 18 years of age and identified by the patient in line with the following definition: “*unpaid, informal providers of one or more physical, social, practical and emotional tasks. In terms of their relationship to the patient, they may be a friend, partner, ex-partner, sibling, parent, child or other blood or non-blood relative*.” [30]. Caregivers could be recruited in a patient/caregiver dyad or independent of a participating patient.

#### Health professionals

Health professionals (*n* = 20 per country) were drawn from the clinical teams in each country. Purposive selection criteria included clinical role (e.g. palliative care doctor, clinical officer, nurse, social worker), service role (patient care, service management), and primary location of role (e.g. community-based, inpatient wards).

#### Policymakers

Policymakers (*n* = 10 per country) were purposively sampled based on their jurisdiction and involvement at a district or national level, and a policy remit of one or a combination of cancer, non-communicable diseases or digital health and technology.

### Recruitment and data collection

#### Patients and caregivers

Clinical staff at recruiting facilities reviewed clinical records to identify potential patient and caregiver participants, introduced the study and referred them to the researchers if they were willing to participate. The patient and caregiver topic guides addressed current interaction with palliative care, current use of technology, and potential and actual benefits and barriers of digital technology. Patient and caregiver information sheets and consent forms were translated by professional translators who are experts in the local language in each country. Translators performed forward translation, back translation and resolved differences through discussion. All interviewers (data collectors) could speak local languages – some interviews took place and switched between languages – but then transcripts were written, back translated and then checked by the interviewer. Another researcher checked the translation and where necessary listened to the audio recording. Differences were resolved through discussion. Some interviews were transcribed by professional transcribers/translators.

#### Health professionals

Participants were identified by clinical leads and facility managers. We used a topic guide that aligned with stages of the data-use conceptual framework: data demand, data collection (e.g. feasibility of digital health approaches to patient-level data collection), data availability, causal elements linked to organisational, technical and behavioural factors influencing data use. Furthermore, we identified how patient-level data reflecting experience and outcomes (obtained using mobile phones) might directly influence their clinical practice and service development [31].

#### Policymakers

Participants were identified and approached by the African Palliative Care Association, and academic and clinical teams in Nigeria, Uganda and Zimbabwe. We developed topic guides which addressed access to, and use of, evidence to inform decision making, preferred mode of data transfer and presentation, frequency of data reporting to inform decision-making on financing of palliative care services, and broader national landscapes for health technology development. Topic guides were iteratively refined. We held meetings weekly to debrief on interviews and we used field notes and reflective forms which were completed by data collectors.

**Fig. 1:**
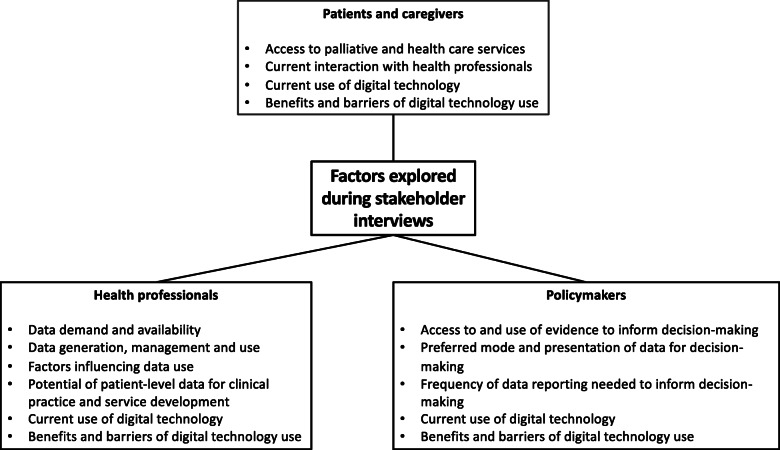
Factors explored during interviews with stakeholders

For all participants, we administered a demographic questionnaire to capture sample characteristics. In terms of data collection and management, all researchers who collected data have over 10 years’ experience of conducting qualitative research. DA and SA are both PhD holders and Lecturers in Sociology at the University of Lagos, they collected data in Nigeria. AD is a social and behavioral scientist at the University of Zimbabwe with a degree in Sociology. She collected data in Zimbabwe. ENab is a palliative care professional and researcher at Makerere university and has a degree in palliative medicine, ENamu is a palliative care professional with doctoral training in health research and is a Lecturer at Makerere University, and ENami is a research manager at African Palliative Care Association and palliative care professional with doctoral training in health research. They all supported data collection in Uganda. All the interviewers have an interest in palliative care and digital technology. Furthermore, ENami has interest in palliative care policy. The interviews were audio recorded and uploaded on the Microsoft OneDrive platform, thereafter we deleted the recording from the audio recorder. Figure 1 summaries factors explored during the interviews with all stakeholders.

### Data analysis

Sample characteristics were descriptively analysed in Stata version 15 [32]. Participant interviews were transcribed verbatim and translated into English (where necessary) before being imported into NVivo version 12 for framework analysis, with all stakeholders’ samples analysed jointly to a single coding frame. This enabled the team to develop an understanding of all stakeholder views for each theme [33]. KBN coordinated data analysis. He developed a coding frame using both deductive and inductive approaches. An initial framework was developed from patient transcripts (*n* = 3), caregiver transcripts (*n* = 3), health professional transcripts (*n* = 3), and policymaker transcripts (*n* = 2) for each country. This coding frame was shared with the full study team (DA and SA in Nigeria, ENab, ENamu and ENami in Uganda, and AD in Zimbabwe). Researchers from each country then applied the framework to a further sample of *n* = 3 transcripts for each stakeholder group to ensure that the frame was applicable and added new codes where necessary. The team then convened for a meeting virtually to compare and discuss the coding framework. Differences were resolved through discussion. Thereafter KBN and MJA merged the coding frames from all teams into one framework which was eventually used to code the remaining transcripts in the dataset. Researchers in each country agreed which transcripts to code to ensure that all transcripts were coded. KBN and MJA checked the coded transcripts and discussed with each country team any issues observed with coding. Thereafter KBN and MJA merged the completed NVivo files into one final file.

**Fig. 2:**
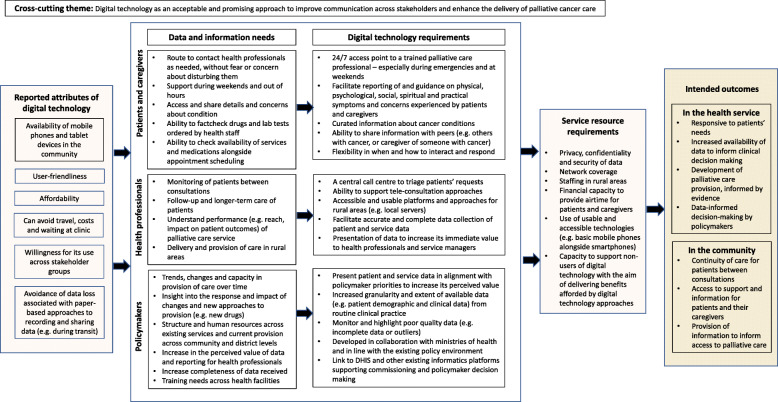
Summary of key findings from stakeholder interviews

Comparative analysis in the framework enabled us to identify common themes as well as country-specific and stakeholder group divergences. A model of the coding frame was developed, and each theme and subtheme given a definition to ensure the internal consistency of each code. Illustrative quotes are reported for each theme, alongside study participant ID and country to demonstrate reporting from across the sample breadth. Following analysis, we adopted a thematic network approach [34] to generate a schematic depicting how principal themes and patterns that emerged in the analysis aligned with the original questions. MJA, BE and KBN developed an initial thematic network which was then adapted through two iterations of feedback to the wider research team. The final thematic network is presented in Fig. 2.

### Ethical considerations

Ethics approvals were obtained from the Institutional Review Boards of University of Leeds (Ref: MREC 18–032), Research Council of Zimbabwe (Ref: 03507), Medical Research Council of Zimbabwe (Ref: MRCZ/A/2421), Uganda Cancer Institute (Ref: 19–2018), Uganda National Council of Science and Technology (Ref: HS325ES) and College of Medicine University of Lagos (Ref: HREC/15/04/2015). The project was aligned with the Medical Research Council good research practice guidelines [35] and H3Africa framework for conducting ethically responsible biomedical research [36].

### Patient and public involvement

Patients with advanced cancer and their caregivers were consulted at the design stage of the study in participating countries, reviewing information sheets, consent forms and topic guides for appropriateness of language. Content of the forms was reworded in response to feedback, probes were suggested that could be adopted to encourage detailed discussions, and described how to articulate the meaning of palliative care in the local context. The study design was also discussed with patients and caregivers, outlining what would be asked of participants in the study. Feedback from discussions prior to the study commencing suggested that patient and caregiver study participants should receive a small token of appreciation for participating in the study. They emphasized the need for compassion towards patients and caregivers who are investing time to participate in the study. It was ensured that participants were provided with transport costs and provided with refreshments during interviews. Furthermore, discussions with patients prior to the start of the study raised concerns around mobility to attend interviews at health facilities verifying the need for planned flexibility in the timing and location of interviews. Alongside guiding the study design and content, non-participant patients with advanced cancer and their caregivers participated in a webinar to discuss dissemination plans for the study, with patients as panellists, steering discussions. Engagement of patients in Zimbabwe regarding dissemination of study findings is ongoing, alongside determining preferred approaches for sharing study findings with participants.

## Results

### Sample characteristics

The majority of patient participants were females (56.45%), married (54.84%), and the most common diagnosis was breast cancer (29.51%) (see Table 1). The majority of patients (82.26%) and caregivers (87.5%) attained at least secondary education. The majority of health care professionals were females (67.80%). Interviews lasted an average of 45 min, ranging from 20 to 90 min.

**Table 1:** Characteristics of study participants (*N* = 195)

Cancer patients (***n*** = 62)	n (%)
Sex	
Female	35 (56.45%)
Male	27 (43.55%)
Mean age years (SD)	51.61 (16.20)
Marital status	
Married	34 (54.84%)
Single	13 (20.97%)
Divorced/Separated	9 (14.52%)
Widow/widower	6 (9.68%)
Education	
Secondary	31 (50.00%)
Tertiary	20 (32.26%)
No education	2 (3.23%)
Primary	9 (14.52%)
Religion	
Roman Catholic	20 (32.26%)
Pentecostal	18 (29.03%)
Anglican	5 (8.06%
Apostolic	4 (6.45%)
Methodist	2 (3.23%)
Seventh Day Adventist	2 (3.23%)
No religion	2 (3.23%)
Other	9 (14.52%)
Cancer type	
Breast	18 (29.51%)
Prostate	8 (13.11%)
Colon/rectum	5 (8.20%)
Cervix	5 (8.20%)
Lymphoma	4 (6.56%)
Liver	3 (4.92%)
Leukaemia	3 (4.92%)
Lung	3 (4.92%)
Ocular Squamous cell carcinoma	3 (4.92%)
Bladder	2 (3.28%)
Pancreas/Oesophagus	2 (3.28%)
Multiple myeloma	2 (3.28%)
Spinal/brain tumour	2 (3.28%)
Kaposi’s Sarcoma	1 (1.64%)
Country	
Nigeria	22 (35.48%)
Uganda	20 (32.26%)
Zimbabwe	20 (32.26%)
**Caregiver participants (*n*** **= 48)**	
Mean age (SD)	37 (13.44)
Sex	
Male	24 (50%)
Female	24 (50%)
Country	
Nigeria	18 (37.50%)
Uganda	15 (31.25%)
Zimbabwe	15 (31.25%)
Education	
Secondary	22 (45.83%)
Tertiary	20 (41.67%)
No education	2 (4.17%)
Primary	4 (8.33%)
Marital status	
Married	23 (47.92%)
Single	22 (45.83%)
Widow	3 (6.25%)
Religion	
Pentecostal	18 (37.50%)
Roman Catholic	12 (25.00%)
Anglican	4 (8.33%)
Apostolic	4 (8.33%)
Methodist	2 (4.17%)
Jehovah witness	2 (4.17%)
No religion	6 (12.50%)
Carer relationship	
Sibling	18 (37.50)
Son/daughter	15 (31.25)
Husband	7 (14.58)
Parent	5 (8.33)
Wife	4 (8.33)
**Health Care Professionals (*n*** **= 59)**	
Sex	
Female	40 (67.80%)
Male	19 (31.20%)
Professional cadre	
Nurses	20 (33.90%)
Doctors	19 (32.20%)
Counsellors/Community Care Providers	11 (18.64%)
Social workers	8 (13.56%)
Clinical Officer	1 (1.69%)
Mean (SD) years’ health professional experience	16.59 (11.13)
Mean (SD) years’ palliative care experience	9.70 (7.52)
Country	
Nigeria	19 (32.20%)
Uganda	20 (33.90%)
Zimbabwe	20 (33.90%)
**Policy Makers (*n*** **= 26)**	
Sex	
Male	12 (46.15%)
Female	14 (53.85%)
Professional background	
Doctor	13 (50.00%)
Nurse	3 (11.54%)
Pharmacist	3 (11.54%)
Health Informatics	2 (7.69%)
Public Health Specialist	2 (7.69%)
Statistician	1 (3.85%)
Social worker	1 (3.85%)
Surveillance Manager	1 (3.85%)
Years’ experience at policy level (SD)	11 (6.29)

### Main findings

Our coding framework identified *four* main themes and these were: i) Acceptability of digital technology; ii) current context of technology use; iii) current vision for digital technology to support health and palliative care, and; iv) digital technologies for the generation, reporting and receipt of data. Table 2 contains a description of these themes and quotes.

**Table 2:** Summary of main themes and quotes from study participants

Theme	Description	Quote
1 Acceptability of digital technology	All stakeholder participants were excited about using digital technology to contact palliative care services.	Quote 1: “*It will be very good, it makes the work easier and faster so whenever there is a problem, I just call or text someone, then the person replies to me than me coming down to the hospital before I need to see the doctor, before our appointment date if there is any problem I will just call the palliative health care service, is there any drug it is very very good, it will actually reduce the stress”* (caregiver: Nigeria). Quote 2: “*We don’t have enough healthcare providers across the country and it’s difficult to get people to go to some of these areas that are a bit far and hard to reach. So mobile technology, it’s where the world is going, we can’t avoid it, I think we should have to adapt it, adapt it to suit the services we can provide”* (health professional: Nigeria).
	Some participants had concerns using technology to contact palliative care services. They were concerned about breach of privacy and confidentiality and that there may be loss of data during transmission. These concerns were mainly raised by caregivers, health professionals and policy makers.	Quote 3: “...*but there must be that privacy; you don’t just share out someone’s information, there should be some control. I expect the health professionals to kept confidentiality”* (caregiver: Uganda). Quote 4: “… *it may interfere with the confidentiality of the health personnel or some kind of data linkages, for example if I text you I am not so sure who will read the message. There some patients who give their phone to other people”* (health care professional: Uganda) Quote 5: “*...There are hackers out there, they access that information, and do we have a way of making sure we also don’t lose that data, the computers or the-even the tablet crashes”* (Policy maker 02–045, Zimbabwe).
	Patients participants were hesitant to use technology for fear of disturbing health professionals, while health professionals were concerned that some patients will call during awkward hours.	Quote 6: “… *though sometimes I want to call but, err, I just feel maybe because it is only one doctor’s number I have so most times when I want to call him I feel maybe I am disturbing him because he is the only one I am calling and sometimes I call him, he doesn’t pick and I just feel maybe I am disturbing him* (Breast cancer patient: Nigeria). Quote 7: “… s*ome, they will call you at 3:00 am ha, because I have had an experience when I was still on the ward a patient called I said ha this is not this is an odd time”* (health care professional: Zimbabwe).
	All stakeholders stated that most health facilities do not open over the weekend or do not have a call centre at night time.	Quote 8: *“...hospice does not have a 24-h help line service to help people like us, that somebody we call and will pick the phone at all time, may be a receptionist, something like that, they don’t have it... so, when you get an attack at night or after 5 or 6 pm and over the weekend, and over the public holidays”* (caregiver: Uganda). Quote 9: “… *like a call centre that is set up. Those guys are trained in palliative care, they can actually try and triage the patient over the phone, I think it’s better for you to go to a 24-h because you are not, definitely not going to be managing the patient over the phone”* (health care professional: Zimbabwe).
2. Current context of technology use	Caregiver and patient participants reported that they use technologies to search for information on the internet about their cancer.	Quote 10: “*...like all my results, all the test I did nobody explained anything to me, I had to try and know what it is using my phone, so I had to google what is the name of this test, it will tell me. So, I know the kind of cancer I am dealing with I know the kind of treatment that is best for me even without my doctor talking to me I already know what am supposed to do, and things am not supposed do”* (breast cancer patient: Nigeria).
	Caregiver participants reported sharing recorded videos with colleagues in a similar situation.	Quote 11: “… *I belong to one group of caregivers and sometimes they upload some videos, share some exercises that are good for especially for people with the lymphedema, so I watch those videos and encourage my patient to do”* (caregiver: Nigeria). Quote 12: *“I used technology, WhatsApp call to communicate with the health professionals and were able to see the patient and also sent videos and photos of the patient sores”* (caregiver of a Kaposi Sarcoma patient: Zimbabwe).
	Caregivers and patient participants contacted palliative care to ask about availability of services, or medication or booking appointment.	Quote 13: “*I think it is useful in any, every kind of situation, when you are just looking for information about your illness, you need an immediate answer from a doctor, whether you need to know the availability of your medication, like thoroughly or even when you need to book an appointment with your doctor. It’s very useful in every kind of situation”* (caregiver: Zimbabwe).
	Caregivers reported using technology in critical situations. Health professionals used technology to make follow-ups about the patient. Participants reported benefits of using technology.	Quote 14: “*More especially if the patient is really very sick, you feel like when you just send text message you feel may be that person has not received the information or she has not known the gravity of the sickness so you personally need to go to the hospital”* (caregiver: Uganda). *Quote 15: “Of course, finding out how is the patient is doing, sometimes if the patient was very sick, may be the patient passed on, so that information is very important, for me to follow up and find out how the patient is doing”* (health professional: Uganda). Quote 16: *“… we also call to confirm if the patient will be at home when we are planning to come for our home visit”* (health professional: Uganda). Quote 17: “… *you find a traffic jam using technology will save time and also at the same time it will save the patient from her pain … you can communicate directly and get answers directly, instead of going to hospice”* (caregiver: Uganda). Quote 18: “S*tress on the main road, like my condition now if you enter any gallop it affects me, I was rolled from this err bumpy road to this place, my pain increased, if it is something that I should be at home and call I don’t think all these pains will increase at least I will be a little bit reserved, than to add to the problem I am having*” (breast cancer patient*:* Zimbabwe). Quote 19: “T*he advantage is that you have all this information in one place and you’re able to refer back to it and you’re able to analyse per period... you can do so many things with that data”* (policy maker: Uganda). *Quote 20: “… it shortens the distance that you have to go to provide services and makes it faster, makes it easier, makes it direct, definitely … it’s possible that there might be a new drug that is come into the market now.....let me give my cancer patient they say it very good for this, and then the cancer patient then may react to the drug, this kind of technology will help him to be able to access the patient......whatever it is that he needs to do, He doesn’t have to wait till the next clinic day to be able to have access to the doctor.....so real time solutions are provided for real time problems”* (policy maker: Nigeria).
	Caregivers and health professionals cited nature of the job and problems with electricity as barriers to using technology.	Quote 21: “… *sometimes you are in a briefing you can’t use phone, you have to keep the phone away from you and all that, or if it’s at work I will definitely want to put my phone on silent so I had to put the phone on silent or I will just leave it in the drawer of the office”* (caregiver: Nigeria). Quote 22: “… *when you are at the party or when you are in at burial or in church... because it doesn’t mean that a health professional is supposed to work 24 h in 360 days all that. You may be in a vehicle, you may be in a place when its noisy, when you call me when am on a busy street, I can’t get my phone out”* (health professional: Uganda). Quote 23: “*You may have distress or concerns with your patient at night and when you call them at such time, your phone may not have airtime or even run out battery...yah”* (caregiver: Zimbabwe). Quote 24: *“… it is our day to day life even if you don’t have airtime that is why they make it easier, in case of emergency you can just borrow airtime, then there is power bank so you can charge your phone*” (caregiver: Nigeria). Quote 25: “*There some that you cannot afford, in some cases where you might not be able to afford, of course ok now for example me I think a smart phone would provide wider range of options and operations.... when you think about the cost compared to a normal phone, and yet most people can afford the normal phone”* (caregiver: Uganda). Quote 26: “… *some applications are a little bit complex, and people don’t grasp to* use” (pancreatic cancer patient: Uganda). Quote 27: “*Because some will be the elderly, and they won’t be able to send the data and the like, but if they are young adults, well it is something that is really good, elderly people, are just able to receive a call and say ‘hello’”* (health care professional: Zimbabwe).
3: Current vision for digital technology to support health and palliative care	Policy makers outlined several things they envisioned in terms of using technology in the near future.	Quote 28: “… *integration of data so that at each level of health care system they should be able to access patients records.....Is that whichever health worker depending on whichever level they are on, you know the structure from community to health facility, district whichever person which is on those different levels should be able to access data on particular things as easy as possible of course depending on the authorization bit”* (policy maker: Uganda). Quote 29: “*...we need easy to use technologies to collect data in the patients’ homes hand held devices is system that is integrated, so that information at patient level in the home can be collected. But that is integrated with for example the pharmacy, because there is a link between care and medications, but that also incorporates all the different aspects of our care palliative care, pain symptoms, psychosocial, spiritual issues”* (policy maker: Uganda). Quote 30: “*Well, there is what is called the DHIS … which is national system, the electronic national system that is used nationally where all facilities are expected to comprehensive report on all the diseases, of course limited to the … number per facility, what they are,,, the diagnosis they are making and all that, so we have that system and it is in the planning for every state of it is a national we have disease”* (policy maker: Nigeria). Quote 31: “… *In the next 5 years or so that we shall be digital and you can get all information on your phone, at the tip or stroke of the button that you can pick information very quickly, so that’s the kind of vision. Also, that you can get information about a particular patient by simply going into the computer, yeah that’s the vision that we are having that everything is going to be digital, very easy and available to anybody. So that you don’t have to come all the way to the facility and tell me about something I can get from a phone”* (Policy maker: Uganda).
4. Digital technologies in the generation, reporting and receipt of data	Health professionals and policy makers stated that digital technology can be used to generate and report data from grassroots to national level, however some health professionals do not appreciate the value of this.	Quote 32: “… *at the grass roots, even when we have these tools or these avenues to capture the data, probably those people, at those points of care, they probably do not understand the need, the importance of data. So, as long as they fill in these other, what they call basic or routine information, they don’t go down to informing other things, like for example, use of substances, use of alcohol, they ignore those. So most of the time, they’re told to return back and complete those blanks”* (Policy maker: Uganda).
	Demands for greater granularity in data to increase its utility in decision making.	Quote 33: “*I don’t think so, just knowing the amount of morphine usage is enough, data should also contain the number of health care workers, are they qualified?, what kind of education level do they have, where did they train, are they able to do home care services, where did they find their patients, and also getting the view of the patient, if I don’t have all that information, I would not say the data I am collecting is adequate and can real inform my decision”* (Policy maker: Uganda)

#### Theme one: acceptability of digital technology

Participants were positive about using digital technology, such as mobile phones. They stated that this is an easier way to engage palliative care facilities especially between consultations, such as making an enquiry. Similarly, some health professionals welcomed the development because it is challenging for patients to access palliative care services due to distance issues and shortages of health professionals especially in rural areas (Quote 1–2). Concerns were also expressed about using digital technology. Participants were concerned that using digital technology may breach privacy and confidentiality (Quote 3–4). In terms of using other forms of technology such as computers, iPads, tablets and laptops, policymakers expressed caution when transmitting data or sharing information to prevent a data breach and or loss of data (Quote 5).

Patients and caregivers could be hesitant to make a phone call because they were concerned that the doctor may be busy and were reluctant to disturb them. Some patients also felt that they can better articulate and explain their concerns or distress to the doctor when meeting face-to-face. Health care professionals were concerned that some patients may call during awkward hours especially when they are not at a duty station. Moreover, most facilities don’t have a central point where you can call including 24/7 facilities. Health care professionals therefore felt that there should be a central point of contact for the patients and caregivers to call if they need help anytime.

#### Theme two: current context of technology use

The context of use of technology clustered around six issues:

i) Patient and caregivers use of technology to search and share information about cancer

Caregiver and patient participants reported that they use technologies to search for information on the internet about cancer. For example, some patients searched for information about traditional herbs and plants that may be used for cancer treatment, some patient participants reported searching for information about the type of cancer they have, its prognosis, the laboratory tests conducted, how to interpret the results and diet. They reported that in most cases clinicians do not provide accurate information about the laboratory tests or prognosis so technology is used to provide this instead (Quote 10). Patients and caregivers also reported sharing information with others in a similar situation (e.g. videos on self-management exercises for cancer patients, home management of symptoms and concerns) through existing patient and caregiver networks or support groups (Quote 11).

ii) Contacting healthcare service

Some caregivers shared their video clips, captured through a mobile phone, with healthcare professionals to show, for example, pressure sores their patient had developed. Health professionals were able to use these videos to determine the next steps in the patient’s care. Digital technology was seen as helpful in situations where patients just want to make enquiries about current availability of a particular service (e.g. whether the radiation machine is working) or booking, confirming, or cancelling an appointment (Quote 13).

iii) Drivers of digital technology use to contact palliative care

Participants reported important and critical situations when digital technology was used in emergency situations, for instance, a patient fainting, bleeding or having uncontrolled pain. In these situations, they could not send a text message and had to call directly. Patient and caregiver participants made calls to seek advice about medication, such as when a patient had taken morphine but had unresolved pain. Digital technologies were commonly used during the night or over the weekend when hospice offices were closed and participants were keen to receive advice and guidance about dealing with side effects of medication or seeking psychological, counselling, and emotional care if the patient or caregiver was distressed (Quote 14).

iv) Health professionals’ use of technology to monitor and follow-up patients

Health care professionals reported that they make calls to follow-up with patients that miss an appointment. They also call patients if they want to check the progress of a new intervention (e.g. medication change) or if they are planning a home visit to make sure the caregiver or the patient is available at home (Quote 15–16).

v) Benefits of using technology

Patient and caregiver participants stated that ease of contacting palliative care services irrespective of location and transport challenges was a benefit. They found it cheaper than visiting in person as it can remove transport costs, prevent unnecessary travel and stress experienced on the road, and avoid long waiting times at the clinic (Quote 17–18). Policymakers described digital technologies as beneficial through collating information or data (e.g. medications prescribed, side effects, drug toxicity) in one place for both monitoring patient responses to the treatment prescribed and visualising data to inform decision making (Quote 19–20).

vi) Barriers of using digital technology

Some caregiver participants reported that the nature of their job or their work environment may prevent them from hearing a call or being able to answer it. Some participants were reluctant to answer a call on a smartphone in a busy street for fear of it being stolen (Quote 21–22). Some participants reported that they have problems with electricity, experiencing frequent black outs and not being able to charge a phone. Participants also described financial barriers, not having money to buy credit to be able to make a phone call, especially at night (Quote 23). Other participants thought that using mobile technology is common and even if there are challenges of credit or airtime, there are ways to borrow and pay when you have the money and there are numerous backup options when a power failure is experienced (Quote 24). Some participants said that other barriers could be the type of phone itself, such as smart phones, which are not affordable and may be quite complex and challenging to operate or navigate (Quote 25–26). Some participants felt that any mobile phone may pose challenges for elderly people (Quote 27).

#### Theme three: current vision for digital technology to support health and palliative care

Policy makers were excited with use of digital technologies in the delivery of palliative care, not only for cancer patients but for health services in general. They reported various existing and emerging policy documents and their vision in the use of mobile technology. Some policy makers reported envisioning systems that integrate all patient-level data, including demographics, clinical records and current treatments to support allocation of resources guided by data (quote 28). Policymakers reported a preference for digital technology approaches that are easy to implement and accessible at all levels of healthcare. They were also keen to ensure that interventions are well-integrated into routine clinical practice using a person-centred approach (Quote 29). Policymakers proposed having several registries collecting and recording data to support decision making at a national level. Such data would be valuable to inform planning and to develop appropriate interventions for cancer patients. Some policymakers hoped for data to be available in all parts of the country and accessible to all stakeholders who require it, supporting generation of a high-quality evidence base (Quote 30). Some policymakers went on to note that they want all data reporting to be through digital technologies in the coming 5 years. This would avoid loss of paper files, ensure patient clinical data is informing decision making and helping data to be accurate, complete and useful for planning services (Quote 31).

#### Theme four: digital technologies for the generation, reporting and receipt of data

i) Generation and reporting of data

Digital technologies were reported as affording advantages over existing approaches to data generation. Existing paper-based approaches to recording and sharing data leads to data loss during transit, particularly from hard to reach areas, and incomplete data. Healthcare professionals were reported as not seeing value in gathering patient data so often leave data fields blank on reporting forms. This leads to data that is not usable for guiding decision making and planning interventions (Quote 32). Digital technologies were in place across all countries, including systems to capture data at the district level (i.e. District Health Information Software (DHIS)) and for the ministries of health gathering data from health facilities nationally to inform decision making and planning (i.e. Health Management Information System (HMIS)). Whilst the HMIS is in use in all countries, captured data on cancer were limited to a few indicators and lacked the granularity to guide cancer and palliative care service planning. Issues with incomplete reporting by health professionals persisted for these systems too, with poor quality data alongside the added barrier of a shortage of health care professionals able to capture and record data diligently.

ii) Utility of existing data

Current information reported to local and national authorities includes a mix of sociodemographic, clinical data (e.g. type of cancer) and service utilisation data (e.g. treatments or interventions received, prescription of morphine), although the breadth of items captured varied considerably across the three countries. Despite more detailed data capture in Uganda, the data still lacked utility. For example, policymakers thought that just reporting the amount of morphine consumed or the number of patients who were in pain is not adequate. Data capture should include details about the number of healthcare workers providing services, what type of services they provide, their training and qualifications, and information about their mentorship needs, in order to design services for cancer patients that are both feasible and sustainable (Quote 33). Furthermore, policymakers in Nigeria and Zimbabwe highlighted the need for more detailed data capture on patients with cancer, such as extending beyond grouping all cancer cases to reporting instances of specific cancer types. This could then guide appropriate and specific approaches, such as prevention strategies, for the most prevalent cancers.

## Discussion

This study identified widespread use of digital technologies across the provision of palliative care services by patients, caregivers and health professionals in Nigeria, Uganda and Zimbabwe. It is the first study to explore preferences for the development of digital technology approaches across all key stakeholders in the SSA region. Both acceptability and reservations about digital health approaches were identified. Preferences and needs for the use of technology were mostly device agnostic, reflecting instead the dynamic of interaction that can be supported through digital technologies such as the frequency of contact and drivers of digital technology use. For policymakers, digital technology approaches feed into a wider vision for the development of data and its availability and use to inform the planning and development of services for patients with cancer.

This study is the first to report consultation across stakeholder groups, including patients, caregivers, and policymakers in three countries in the SSA region. Involvement of key stakeholders and potential users of technologies is crucial for health technology development [37] and increasing understanding of the context in which they are to be implemented [38]. Previous research has highlighted the need to capture the needs of end-user perspectives to inform the development and evaluation of digital health approaches for palliative care in SSA [17], and willingness of health professionals to develop digital health approaches for palliative care in Nigeria [39]. This study addresses these gaps in knowledge, deriving a set of 15 requirements (as outlined in Fig. 2) that align data and information needs of stakeholders with digital health intervention components for palliative cancer care in SSA. These 15 requirements are device-and system-agnostic and propose linkages between stakeholder needs, the application of technology, and intended patient-level and health service outcomes. This is a first step in explicating these relationships with subsequent research required to align the modelling, development and evaluation of digital health interventions with findings from this research, alongside determining the mechanisms of action across stakeholder needs, digital technologies, and intended outcomes from their implementation. The insights gathered from multiple stakeholders in this study may inform the development of digital technology approaches at different levels of the health care system, across the patient, care team (including health professionals and caregivers), organisation, and wider environment (i.e. health care purchasers, service commissioners, policymakers) perspectives [40].

Palliative cancer care is vastly underdeveloped in the context of SSA [41]. Stakeholders at all levels of the health systems of the three participating countries see potential for digital health technologies to support the development and reach of palliative care services. The evidence of benefit from the use of digital technology to support, for example, symptom management and clinical decision-making in the context of cancer care is developing [42]. However, similar to knowledge of feasible and effective uses of digital health strategies in LMICs broadly, the evidence base is weak [43]. This study develops the evidence on user preferences for the development and implementation of digital technologies for palliative cancer care. A crucial next step will be to further refine an understanding of the key mechanisms through which digital health technologies can support service delivery and patient outcomes in the context of palliative cancer care in SSA. The development and testing of resultant digital health interventions should seek to identify, recruit and retain the same level of stakeholder engagement as detailed in this study, supporting a better understanding of their implementation. For example, a recent process evaluation framework for mHealth interventions outlines exploring the major active components of the intervention, but also the technology of the intervention, cultural congruence, task shifting, and, crucially in a rapidly developing area, unintended consequences [44]. There are also existing approaches that can be used to refine and develop resulting digital health interventions, such as Multiphase Optimization Strategy (MOST) [45], a novel framework for optimising intervention delivery strategies using a factorial design. This may provide a relatively efficient approach to evaluating iterative development and refinement of digital health approaches whilst requiring relatively small sample sizes; an approach that has been used successfully in developing a strategy to facilitate retention and viral suppression among people living with HIV/AIDS in Tanzania [46]. Across the stakeholder groups included in this study, policymakers have the greatest capability to catalyse development of digital health for palliative cancer care in SSA. Working with donors and private industry, SSA governments are well-placed to develop common standards for developing and implementing interoperable and user-friendly technologies to strengthen health care systems (e.g. through the development of open architecture approaches to digital health systems and integration of sustainable and well-evidenced technologies into cancer control plans) [47]. Policymakers are also best placed to address a key concern raised by participants; privacy and confidentiality of data shared via digital technology approaches. Governments and multinational bodies are best placed to define and demand appropriate digital data governance checks and balances, with emerging governance frameworks available to guide practice [48]. This is not an issue specific to cancer and palliative care and needs to be considered broadly across health and care provision in LMICs. Similarly, a wider consideration will be ensuring inequity does not arise from pursuing digital health approaches. Despite widespread mobile networks across SSA, digital divides by gender and socioeconomic strata persist, costs associated with mobile phones remain high, and people from lower incomes and levels of education are less able to access and use a mobile phone [49, 50]. There remains a need to advocate for greater investment in known enablers of digital inclusion, including infrastructure, affordability, consumer readiness and availability of content and services [24]. Our findings emphasised stakeholders’ preferences for leveraging usable and accessible technologies for palliative care digital health interventions (e.g. basic mobile phones alongside smartphones) and the need to consider capacity to support non-users of digital technologies. Recent principles have emphasised that current approaches to digital technology design, development, implementation and evaluation may be problematic for the worst off [51]. Continued stakeholder engagement in the development of digital technologies should ensure participation of the marginalised in programmes to ensure an understanding of sociotechnical complexities of implementation. Alongside this, augmentation of approaches need to be explored to ensure those who opt not to use digital technologies are not inadvertently disadvantaged.

### Strengths and limitations

To our knowledge this is the first study to explore perspectives on use of digital technology for the delivery of palliative cancer services in SSA from a diverse group of stakeholders. We recruited participants from three different countries with diverse cultures, religious beliefs and tribes in West Africa (Nigeria), East Africa (Uganda), and Southern Africa (Zimbabwe). We had a large sample across the three nations which was sufficient to reach data saturation and consisted of a diverse group of participants in each stakeholder group. Our purposive sampling frame was largely achieved. This included policymakers who were recruited from both ministries of health and advocacy organisations involved in policy formulation and planning at national and regional levels relating to non-communicable diseases and digital health. Limitations of this study are that findings may not be applicable to settings beyond the three included nations where data was collected. This is particularly the case for data from Nigeria which was collected in Lagos State. Nigeria has multiple states with several tribes, therefore data from Nigeria may not be nationally representative. While differences across countries were not prominent in the findings of this study, we would anticipate that context will have an increasing influence in the subsequent development and implementation of digital health interventions. For example, variation in resources, capacity and provision to deliver palliative care services, and the country-specific policy context will need to be explored further in future research. These may be better understood through continued stakeholder engagement which should continue in subsequent stages; it is an essential principle for successful digital health initiatives, particularly when planning for scale and integration [52]. Furthermore, future research will need to extend beyond cancer care to consider multiple disease groups to guide a comprehensive digital strategy for the diagnosis and management of chronic diseases for improved health provision in Africa [53]. We did not provide feedback of our findings to study participants, however, as highlighted in the PPI section, we have been feeding findings back to wider groups representing the interests of key stakeholders who are guiding wider dissemination.

## Conclusion

This work presents a foundation to guide the development of digital health interventions for the delivery of palliative care for cancer patients in SSA. Through stakeholder engagement we identified the design, practical, and contextual challenges to optimise potential for success. A set of 15 requirements were derived from our data that can be used to inform the development of digital health approaches for palliative cancer care in SSA. We identified needs and preferences for digital technology approaches from our stakeholders that can act as user requirements to be taken into consideration when planning and designing digital health interventions and appropriate methods of evaluating their effectiveness in the delivery of cancer palliative care in SSA. This will also inform subsequent implementation and rollout of digital health approaches in SSA, as part of efforts to achieve priorities in global palliative care policy and universal health coverage.

## Supplementary Information

**Additional file 1.**

## Data Availability

The authors have full control over the primary data for this study. The data analysed in this study are stored online in OneDrive folder at the University of Leeds, King’s College London, Makerere University, African Palliative Care Association, University of Zimbabwe and University of Lagos. As per the ethical committee approval from all the countries involved, this dataset is subject to ethical restrictions, and informed written consent of study participants does not include the publication of raw data in terms of interview manuscripts.
