# Supplementary material for: Stakeholder perspectives and requirements to guide the development of digital technology for palliative cancer services: a multi-country, cross-sectional, qualitative study in Nigeria, Uganda and Zimbabwe
Source: BMC Palliat Care. 2021 Jan 4;20:4. doi: 10.1186/s12904-020-00694-y (PMC7784352; doi:10.1186/s12904-020-00694-y)
Supplement: Supplementary file 1 — Additional file 1. [file 12904_2020_694_MOESM1_ESM.docx]

**Consolidated criteria for reporting qualitative studies (COREQ): 32-item checklist**

Developed from:

Tong A, Sainsbury P, Craig J. Consolidated criteria for reporting qualitative research (COREQ): a 32-item checklist for interviews and focus groups. *International Journal for Quality in Health Care*. 2007. Volume 19, Number 6: pp. 349 – 357

**YOU MUST PROVIDE A RESPONSE FOR ALL ITEMS. ENTER N/A IF NOT APPLICABLE**

| **No. Item** | **Guide questions/description** | **Reported on Page #** |
| --- | --- | --- |
| **Domain 1: Research team and reﬂexivity** |  |  |
| *Personal Characteristics* |  |  |
| 1. Interviewer/facilitator | Which author/s conducted the interview or focus group? | Methods: page 8-9 |
| 2. Credentials | What were the researcher’s credentials? E.g. PhD, MD | pages 1, 8, 9 |
| 3. Occupation | What was their occupation at the time of the study? | Methods: Page 8-9 |
| 4. Gender | Was the researcher male or female? | N/A |
| 5. Experience and training | What experience or training did the researcher have? | Methods: page 8-9 |
| *Relationship with participants* |  |  |
| 6. Relationship established | Was a relationship established prior to study commencement? | N/A |
| 7. Participant knowledge of the interviewer | What did the participants know about the researcher? e.g. personal goals, reasons for doing the research | N/A |
| 8. Interviewer characteristics | What characteristics were reported about the interviewer/facilitator? e.g. Bias, assumptions, reasons and interests in the research topic | Methods: Page 8 |
| **Domain 2: study design** |  |  |
| *Theoretical framework* |  |  |
| 9. Methodological orientation and Theory | What methodological orientation was stated to underpin the study? e.g. grounded theory, discourse analysis, ethnography, phenomenology, content analysis | Methods: Framework analysis page 9 |
| *Participant selection* |  |  |
| 10. Sampling | How were participants selected? e.g. purposive, convenience, consecutive, snowball | Methods: Purposive selection criteria page 7 |
| 11. Method of approach | How were participants approached? e.g. face-to-face, telephone, mail, email | Methods: page 7 |
| 12. Sample size | How many participants were in the study? | Methods: Page 6  Results: Page 10  Table 1 (supplementary file) |
| 13. Non-participation | How many people refused to participate or dropped out? Reasons? | Methods: N/A |
| *Setting* |  |  |
| 14. Setting of data collection | Where was the data collected? e.g. home, clinic, workplace | Methods: page 6-7 |
| 15. Presence of non-participants | Was anyone else present besides the participants and researchers? | N/A |
| 16. Description of sample | What are the important characteristics of the sample? e.g. demographic data, date | Results: Tables 1, figure 1 (supplementary files)  page 11 |
| *Data collection* |  |  |
| 17. Interview guide | Were questions, prompts, guides provided by the authors? Was it pilot tested? | Methods: Page 7-8 |
| 18. Repeat interviews | Were repeat interviews carried out? If yes, how many? | N/A |
| 19. Audio/visual recording | Did the research use audio or visual recording to collect the data? | Methods: page 9 |
| 20. Field notes | Were ﬁeld notes made during and/or after the interview or focus group? | Methods: page 8 |
| 21. Duration | What was the duration of the interviews or focus group? | Methods: page 9 |
| 22. Data saturation | Was data saturation discussed? | N/A |
| 23. Transcripts returned | Were transcripts returned to participants for comment and/or correction? | N/A |
| **Domain 3: analysis and ﬁndings** |  |  |
| *Data analysis* |  |  |
| 24. Number of data coders | How many data coders coded the data? | Methods: pages 9-10 |
| 25. Description of the coding tree | Did authors provide a description of the coding tree? | N/A |
| 26. Derivation of themes | Were themes identiﬁed in advance or derived from the data? | Methods: deductive and inductive: page 9 |
| 27. Software | What software, if applicable, was used to manage the data? | Stata & NVivo  9 |
| 28. Participant checking | Did participants provide feedback on the ﬁndings? | page 21 |
| *Reporting* |  |  |
| 29. Quotations presented | Were participant quotations presented to illustrate the themes/ﬁndings? Was each quotation identiﬁed? e.g. participant number | Results: Table 2 |
| 30. Data and ﬁndings consistent | Was there consistency between the data presented and the ﬁndings? | Relationship to existing knowledge  Discussion: Page 18 |
| 31. Clarity of major themes | Were major themes clearly presented in the ﬁndings? | Results: 12-17 |
| 32. Clarity of minor themes | Is there a description of diverse cases or discussion of minor themes? | Discussion: 18 |

**Once you have completed this checklist, please save a copy and upload it as part of your submission. When requested to do so as part of the upload process, please select the file type: *Checklist*. You will NOT be able to proceed with submission unless the checklist has been uploaded. Please DO NOT** **include this checklist as part of the main manuscript document. It must be uploaded as a separate file.**
